# Quantifying the immediate computational effects of preceding outcomes on subsequent risky choices

**DOI:** 10.1038/s41598-020-66502-y

**Published:** 2020-06-18

**Authors:** Hayley R. Brooks, Peter Sokol-Hessner

**Affiliations:** Department of Psychology, University of Denver, Denver, CO USA

**Keywords:** Computational models, Reward, Human behaviour

## Abstract

Forty years ago, prospect theory introduced the notion that risky options are evaluated relative to their recent context, causing a significant shift in the study of risky monetary decision-making in psychology, economics, and neuroscience. Despite the central role of past experiences, it remains unclear whether, how, and how much past experiences quantitatively influence risky monetary choices moment-to-moment in a nominally learning-free setting. We analyzed a large dataset of risky monetary choices with trial-by-trial feedback to quantify how past experiences, or recent events, influence risky choice behavior and the underlying processes. We found larger recent outcomes both negatively influence subsequent risk-taking and positively influence the weight put on potential losses. Using a hierarchical Bayesian framework to fit a modified version of prospect theory, we demonstrated that the same risks will be evaluated differently given different past experiences. The computations underlying risky decision-making are fundamentally dynamic, even if the environment is not.

## Introduction

Prospect theory is conceivably the most successful recent theory in risky decision-making^1^. The theory is founded on the idea that people evaluate options relative to “the past and present context of experience” (p. 277)^1^. While this central insight led to two of the most successful components of prospect theory, the reflection effect in risk aversion (diminishing marginal utility of values expressed as concavity for gains and convexity for losses) and loss aversion (overweighting losses compared to gains of the same magnitude), it is unclear how recent events *quantitatively* influence risky monetary decision-making, moment-to-moment. We sought to quantify how recent events (e.g. previous outcomes) influence choices by analyzing a large dataset of risky choices in the presence of feedback compiled from four studies^2–5^.

Laboratory and field studies have suggested that recent events shape the subjective value of risky choice options^6–9^ and actions^10^. The directionality and mechanism supporting these effects are unclear as some studies of risky decision-making in the presence of feedback have found that previous gains, relative to losses, lead to less risk-taking^6,10^ or a mixture of less and more risk-taking^7–9,11^, discrepancies that may be related to how and when outcomes are realized, or paid^12^. Theories that have sought to specify the directionality of the effect of feedback on risky choice have not described the size of the effect, its characteristics (e.g. how far it extends in time) or the precise decision processes that would be altered by feedback^13^. Interpreting these previous findings is complicated by the fact that almost all of these studies included design features intended to elicit temporal context effects (e.g. explicitly-signaled contexts, displays with cumulative elements) and thus may have strengthened, or even created, dynamic context effects. While such an approach identifies an upper bound on the possible presence and magnitude of context effects, such effects may not exist in less context-focused environments. Many more studies with trial-by-trial feedback have not analyzed or discussed the effects of feedback on risk-taking^2–5,14–24^. We thus sought to examine whether, and to what extent, context affects risky decision-making in a setting with no intentional temporal structure. If found, this would establish that the effect of recent events on risky choices is not merely elicited when designs encourage it, but rather that it is part of the fundamental computations underlying risky decision-making.

“Risky decision-making” here refers to decisions with choice options characterized by known values and probabilities^25^. In such a setting, all choice attributes are explicit and lack temporal dependency (i.e. outcomes or choice attributes on previous trials are unrelated to subsequent trials). Indeed, in the studies compiled for the current analysis, all participants passed comprehension quizzes confirming their knowledge of the probabilities and values in the experiments. That there are no probabilities or values to learn or update means that if participants try to do so, they cannot improve and may instead impair their performance. If a dependency is observed, it would establish a fundamental, robust, and unappreciated role for context in affecting subjective value and/or action under risk.

**Figure 1:**
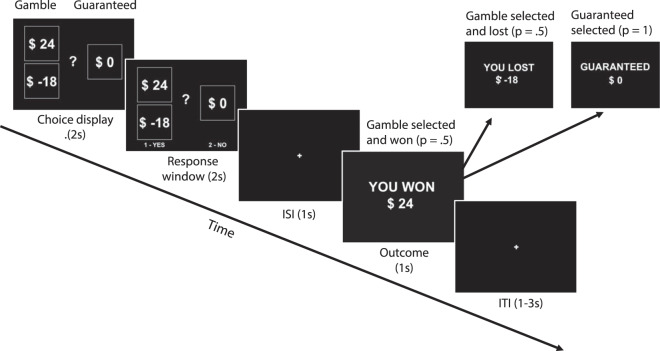
Risky monetary decision-making task structure. Across all four studies^2–5^ compiled for the current analysis, participants made a series of choices between risky and safe options. Every trial featured a risky option with two possible outcomes, each received with 50% probability if that option was selected, and a safe, or guaranteed option received with 100% probability if selected. All choices were followed by outcomes prior to the next choice appearing on the screen. See Supplementary Material and Table S2 for details on the minor variations across studies.

Here, we analyzed a large dataset of risky choice behavior collated across four studies. These studies used an identical risky decision-making task, lacking in temporal dependency, in which participants chose between a risky gamble and a smaller, guaranteed alternative on each trial and received feedback following each trial (see Fig. 1 and Methods for task details)^2–5^. Here, we use “gambling” and “risk-taking” interchangeably to refer to when participants choose the risky option over the guaranteed alternative. First, we fit mixed effects logistic regression models to the data to examine which previous and current choice attributes and events influence risk-taking. We then used a hierarchical Bayesian framework to fit a modified prospect theory model, simultaneously capturing individual differences and group-level effects across a large sample of participants to identify whether and how temporal context influences the processes underlying risky decision-making. This model captures several qualitatively distinct and dissociable processes that contribute to risky choice behavior: loss aversion, risk aversion, choice consistency, and decision bias (see below for more details on each). For each individual, we estimated baseline values for these four valuation and decision processes, and the extent to which each of these processes changed as a function of recent events. See Methods section and Supplementary Material for more details.

## Results

First, we fit mixed effects logistic regression models regressing binary choice (choice of the gamble = 1, guaranteed alternative = 0) on a variety of variables, and second, we fit a hierarchical Bayesian model of the underlying nonlinear processes.

### Mixed effects logistic regression models

We fit mixed effects logistic regression models to the risky choice data using the “lme4” package in R (R version 3.5.0; “lme4” version 1.1–21)^26^. For this analysis, we discuss three types of recent events: previous outcome (the value in dollars of the outcome on the previous trial), previous decision (gamble or guaranteed, coded as 1 for gamble and 0 for guaranteed), and mean expected value (EV) of the previous choice options (the mean of the safe and risky options on the previous trial; see Methods). All mixed effects logistic regression models included binary choice as the binomial outcome variable and both a constant and current choice options (the dollar values of the risky gain, risky loss, and guaranteed alternative) as predictor variables (e.g. choice_*t*_ ~ *β*_0_ + *β*_1 _× risky gain_*t*_ + *β*_2 _× risky loss_*t*_ + *β*_3_ × guaranteed_*t*_ + *β*_4_ × outcome_*t-1*_). The constant was modeled as a random effect and the choice options were modeled as fixed effects. Each model varied only by the type of recent events included as additional predictor variables (for reliable convergence all were modeled as fixed effects). See Supplementary Material and Table S3 for lme4 code for all models.

Across all mixed effects models discussed in this analysis, current choice options had consistent, significant effects on binary choice, such that larger potential gains led to more risk-taking and larger potential losses or guaranteed alternatives led to less risk-taking. Hereafter, we only discuss the additional effects of recent events on risk-taking, controlling for effects of current options. See Supplementary Material and Table S3 for full regression results, including the effects of current choice options.

To first examine which types of recent events had the strongest effects on subsequent risk-taking, we performed three separate regressions (models 1–3), each featuring one of the following: the previous outcome (model 1), the previous decision (model 2), or the mean EV of the previous choice option to capture the average magnitude of the previous trial (model 3). Because the number of parameters in each of these models was identical, to compare models, we used log-likelihood values, or the degree to which each model produced choice likelihoods that reflected the actual choices on each trial. The best-fitting model had the highest (least negative) log-likelihood value.

We found that previous outcomes (model 1) had a significant, negative influence on binary choice, such that as outcomes on the previous trial increased in magnitude, risk-taking on the current trial decreased (*β* = -0.03 (0.003), *p* < 2 × 10^−16^). This model (model 1, log-likelihood = -10,638.5) outperformed models that, instead of the previous outcome amount, featured previous decisions (model 2, log-likelihood = -10,682.7) or the mean EV of the previous choice options (model 3, log-likelihood = -10,669.2). When all three regressors (previous outcomes, decisions, and mean EV) were pit directly against each other by including them in the same model, model 4, previous outcomes predicted risk-taking on the current trial beyond previous decision and mean EV of the previous choice options (previous outcome: *β* = -0.03(0.004), *p* = 4.08 × 10^−15^; previous decision: *β* = -0.04(0.02), *p* = 0.06; mean EV of previous choice options: *β* = -0.002(0.006), *p* = 0.78).

**Figure 2:**
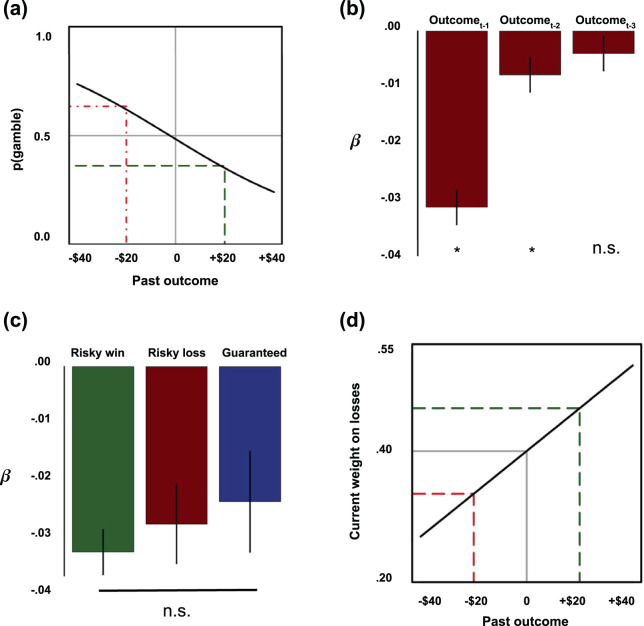
Effects of previous outcomes on current risky choice. (**a**) Visualizing the effect size of previous outcomes of -$20/+$20 on the current choice, assuming indifference on the current trial (model 1). (**b**) Estimates of the effect of past outcomes up to three trials back (t-1, t-2, t-3) on risk-taking on the current trial in model 5 (errors bars represent standard errors). (**c**) Testing whether the effect of previous outcomes varies by type of previous outcome (risky gain, risky loss, or guaranteed alternative) in model 7 (errors bars represent standard errors). No pairwise comparisons were significant (Wald’s Test, all p’s > 0.31), and model 7 was not significantly better than model 1, which collapsed across outcome types (likelihood ratio test, p = 0.56). (**d**) Visualizing the effect size of previous outcomes of -$20/+$20 on the current weight of losses (model 9).

To illustrate the effect size of past outcomes on a given choice, assuming that the participant was indifferent between the current choice options (i.e. the value of the gamble and guaranteed alternative currently under consideration were equal), the estimates from model 1 indicate that the probability of choosing the risky option following a gain of +$20 was 35%, while that following a loss of -$20 was 65% (Fig. 2a), a 30% difference in the probability of risk-taking.

Having established that the effect of previous events on current binary choices was most clearly accounted for by previous outcomes, we subsequently sought to establish the characteristics of that effect over time, valence, and outcome type in models 5–8. First, we examined how far the effect of previous outcome extended over time. In model 5, we regressed binary choice onto the outcome one trial back (t-1), two trials back (t-2), and three trials back (t-3). We found that the negative effect of previous outcomes declined over time, such that it was significant at one and two trials back, but not three trials back (outcome_t-1_: *β* = -0.03(0.003), *p* < 2 × 10^−16^; outcome_t-2_: *β* = -0.008(0.003), *p* = 0.01; outcome_t-3_: *β* = -0.004(0.003), *p* = 0.19; Fig. 2b).

Next, in model 6, we tested whether the previous outcome effect was due to the outcome amount or simply the outcome valence, as previous outcomes included gain, zero, and loss amounts. Regressing binary choice onto regressors for previous outcome amount and for previous outcome valence (modeled as +1 for gains, 0 for zero, and -1 for losses) in model 6, we found that the amount of the previous outcome predicted risk-taking on the current trial beyond the valence of the previous outcome (previous outcome amount: *β* = -0.04(0.005), *p* < 5.95 × 10^−14^; previous outcome valence: *β* = 0.08(0.04), *p* = 0.08).

Finally, we examined whether the previous outcome effect differed as a function of outcome type (gain, loss, or guaranteed). Differences on the basis of outcome type could arise from differences between gains (risky and safe) and losses due to loss aversion^27,28^, or differences between risky outcomes (gain and loss) and guaranteed outcomes due to possible expectation-based learning processes (for example, processes reflecting prediction errors^29–31^) as well as more complex interactions between valence and uncertainty. In model 7, we thus regressed binary choice onto three separate regressors, one each for previous risky gain outcome amount, previous risky loss outcome amount, and previous guaranteed outcome amount. All three regressors were unsurprisingly significant, replicating the overall effect of previous outcomes on binary choice (previous gain: *β* = -0.03(0.004), *p* < 2 × 10^−16^; previous loss: *β* = -0.03(0.008), *p* = 0.0003; previous guaranteed: *β* = -0.02(0.009), *p* = 0.005). Furthermore, these three coefficients were not significantly different from one another as tested in two ways. First, model 7 (with separate coefficients for previous risky gain, risky loss, and guaranteed outcomes) did not perform significantly better than model 1 (with a single previous outcome amount regressor; because model 1 is a nested version of model 7, we used the likelihood ratio test; LRT statistic = 1.17, *df* = 2, *p* = 0.56). Second, estimates for the effects of previous gain, loss, and guaranteed outcomes in model 7 were not significantly different from one another in any of the three possible pairwise comparisons (Wald tests, all three *p*’s > 0.31; Fig. 2c). Together, these findings demonstrate that the effect of previous outcomes on the current binary choice did not differ by outcome type.

For an alternative approach to the analysis of outcome type, in model 8, we regressed binary choice on two regressors that represented outcome amount separately by valence only (with regressors for previous gain amount collapsing across risky and guaranteed outcome amounts, and previous loss amount), and included a separate term for previous risky versus guaranteed choices (coded +1/−1, respectively, as in the other models) which was interacted with previous gain amount. Model 8 similarly identified significant effects of previous gain and loss amounts (previous gain amount: *β* = -0.03(0.005), *p* = 3.22 × 10^−8^; previous loss amount: *β* = -0.04(0.009), *p* = 5.49 × 10^−5^) that were not significantly different from each other (Wald test, p = 0.44). Model 8 also replicated model 4’s finding of a weak effect of previous choices (*β* = -0.05(0.02), *p* = 0.06) when accounting for previous outcomes. Finally, the interaction of previous gain outcome amount with previous risky versus guaranteed choices was not significant (*β* = -0.0009(0.005), *p* = 0.86). Model 8’s results are consistent with models 6 and 7 in identifying that the effect of previous outcomes does not significantly vary by the valence of the outcome or by the type of previous outcome, but is instead best described as an effect of the previous outcome amount, whatever its type or valence.

Thus far, we established that the effect of previous outcome on the current binary choice was short-lasting (Model 5), driven by outcome amount (Model 6), and did not differ by outcome type or valence (Models 7 and 8).

All of the above effects are value-independent in that recent events directly shift the probability of choosing the risky option. However, it is unclear whether recent events may additionally influence valuation of the current choice options. To test whether previous outcomes influence how individuals assess the value of the options on the current trial, in model 9 we regressed binary choice onto previous outcome and included three interaction terms between previous outcome and each of the current choice options (risky gain, risky loss, and guaranteed). While the value-independent effect of previous outcomes on the current binary choice remained (*β* = -0.46(0.21), *p* = 0.03), we additionally found that the outcome on the previous trial increased the weight put on the potential losses (outcome_t-1_ × risky loss_t_: *β* = 0.1(0.03), *p* = 0.002), but not potential gains (outcome_t-1_ × risky gain_t_: *β* = 0.01(0.04), *p* = 0.8) or potential guaranteed alternatives (outcome_t-1_ × guaranteed_t_: *β* = -0.06(0.08), *p* = 0.5). To illustrate the effect size of past outcomes on the valuation of subsequent potential losses, the weight placed on potential losses after an outcome of + $20 was 0.46, or 142% of the weight on losses after an outcome of -$20, which was 0.33 (Fig. 2d).

### Hierarchical Bayesian estimation

Mixed effects logistic regressions allow us to detect individual- and group-level differences for linear processes, but we know that some risky decision-making processes such as risk aversion are not linear^2–5,21,32^.

To address this, for the second analysis we used hierarchical Bayesian estimation, an approach that allowed the fitting of all of the decision-making data at once while simultaneously estimating both individual- and group-level parameters of nonlinear models (like prospect theory) previously shown to fit these data well^2–5,10,21,32–34^. We used Markov-Chain Monte Carlo (MCMC) sampling techniques in a hierarchical Bayesian framework (using “rstan” version 2.17.3)^35^ to fit a modified version of a 4-parameter prospect theory-inspired model (Prospect Theory Plus, PT + )^10^. PT + captured four distinct decision-making processes: risk aversion (*ρ*), loss aversion (*λ*), choice consistency (*μ*), and decision bias (*db*), assuming a linear probability weighting function. Using this Bayesian framework, we estimated both the group-level distribution and individual values for each of the four PT + parameters. We also modeled the change in each of these four parameters over time by altering PT + to include four additional updating parameters (four *δ*^*θ*^ terms, i.e. *δ*^*ρ*^, *δ*^*λ*^, *δ*^*μ*^, *δ*^*db*^). The updating parameters controlled how much previous decision outcomes shifted each of the four original PT + parameters on the subsequent trials. Positive (negative) values of *δ*^*θ*^ would indicate that *θ* increased (decreased) as previous outcomes increased, while a zero value of *δ*^*θ*^ would indicate no net adjustment of *θ* by previous outcomes. Because PT + consists of value-dependent parameters (*ρ*, *λ*), a value-independent parameter (*db*), and an intermediate parameter linking value and action (*μ*), we were able to simultaneously detect the effects of context on linear and non-linear action- and valuation-related decision-making processes. See Methods for the complete modeling procedure.

MCMC estimation procedures produce “chains” of sampled parameter values, in proportion to their likelihood. Using Stan (“rstan” version 2.17.3)^35^, we ran twenty chains of 10,000 samples each, discarding the first 5,000 samples of each chain as a burn-in period, resulting in 100,000 samples. Priors were selected to be as uninformative as possible and were normal, uniform or Cauchy distributions, described in more detail in Table S1. Each of the twenty chains converged on similar distributions of parameter values (mean Rhat for group-level mean parameters = 1.002, range = 1.0003–1.0034; ideal = 1). The total number of effective samples for each of the group-level mean parameters were *ρ* = 10,782, *λ* = 100,000, *μ* = 100,000, *db* = 12,783, *δ*^*ρ*^ = 6,292, *δ*^*λ*^ = 13,003, *δ*^*μ*^ = 12,594, and *δ*^*db*^ = 6,592.

First, we examined mean values and 95% confidence intervals for each of the baseline group-level parameter estimates for risk aversion, loss aversion, choice consistency and decision bias. In our sample, participants were risk averse for gains and risk seeking for losses (*ρ* = 0.65, 95% CI = [0.58 0.73]), were mildly loss averse (*λ* = 1.57, 95% CI = [1.44 1.71]), were consistent in their choices for the risky option (*μ* = 22.2, 95% CI = [18.9 26.0]), and had a bias to gamble (*db* = -0.58, 95% CI = [-0.68 -0.47]), consistent with others’ findings^2–5,10,21,32^.

**Figure 3:**
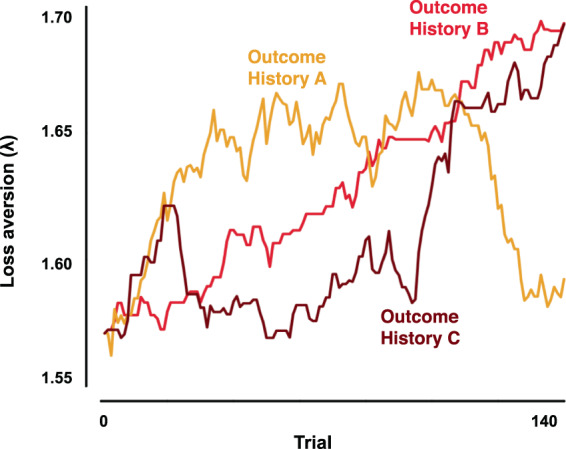
Representative moment-to-moment changes in the loss aversion parameter (*λ*) for a single hypothetical individual as a function of three different sets of outcome experiences ((**A**) yellow, (**B**) crimson, and (**C**) dark red lines), illustrating that the same person will have different preferences, and therefore make different choices, if they have had different recent experiences. The initial loss aversion parameter value (1.57) and the value of *δ*^*λ*^ (0.01) used for this effect size illustration are the group mean values. Note that any given participant’s initial loss aversion or updating parameter values may have been higher or lower than these (see Results for confidence intervals). Outcome histories used here are real and were experienced by three different participants^2^.

Next, we tested whether each of the four parameters updated as a function of previous outcomes. Examining the mean values and 95% CIs for each of the four group-level mean updating parameters, we found that large positive previous outcomes increased loss aversion (*δ*^*λ*^ = 0.013, 95% CI = [0.003 0.02]), consistent with our finding from the linear mixed effects logistic regression analyses that large previous outcomes increased the weight put on potential losses (see above, model 9). We also found that large positive previous outcomes increased consistency across binary choices (*δ*^*μ*^ = 0.08, 95% CI = [0.05 0.10]) and reduced the bias to gamble (*δ*^*db*^ = 0.03, 95% CI = [0.02 0.05]). We found no effect of previous outcomes on risk aversion (*δ*^*ρ*^ = 0.005, 95% CI = [−0.01 0.02]). The variation identified in these confidence intervals means that for identical previous outcomes, different people will react differently. More importantly, the finding of any effect of recent outcomes also means that if otherwise initially-similar people have different experiences, they will subsequently make systematically different risky choices. Figure 3 illustrates this effect by plotting loss aversion over time for one individual experiencing three different histories of outcomes. Risky choice behavior is not just about the individual decision-maker and their choice preferences, but also their past experiences.

## Discussion

Here, we have quantified the consistent and strong effects of recent outcomes on subsequent risky choices. We have demonstrated that the size of the monetary amounts received in previous outcomes has a negative relationship with current risky choices, the effect is short-lasting and linear across outcome types, and recent events appear to have value-independent and value-dependent effects on current actions. These findings are especially notable given the absence of explicit manipulations or design features encouraging integration of previous events with current choices, and the explicit, learning-free setting featuring fully-known values and probabilities. This differs from studies (e.g. on the decision by sampling theory^36–40^ or others^7–11^) that are designed around contextual influence. That risk-taking changes as a function of contextual manipulations in those studies likely reflects both the intrinsic contextual sensitivity of risk-taking as well as the success of the experimental manipulations, but it is unclear how much of the overall effect is aligned with which. We provide strong evidence here that risky monetary decision-making is in fact fundamentally dynamic, even when the environment is decidedly not.

While many dynamic behaviors are well-explained by learning, it is not a compelling explanation here for two reasons: the task, and participants’ reports. First, all monetary values in the task were explicitly shown on every trial and detailed instructions explicitly communicated that probabilities were stable (and simple, being either 100% or 50% in all cases) across all trials. This means that it is unclear what could have been learned in the first place as no elements of the task were hidden, implicit, or otherwise unknown at any time. Second, all participants completed quizzes prior to data collection directly assessing comprehension of the explicit, non-dynamic nature of all task elements, making it unlikely that any of them went on to try to learn about any of these elements. Thus, the major remaining possible explanations for the observed dynamic effects are shifts in the processes underlying valuation and action.

We reported effects of previous outcomes on three of the four decision processes that we modeled (loss aversion, choice consistency, and decision bias). It is important to note that there are additional decision-making processes not included in our model that previous outcomes may influence on a trial-by-trial basis (e.g. probability weighting)^41^. Future research should address additional decision-making processes to more completely identify which processes are affected by previous events and the magnitude and characteristics of that influence.

 The strong effects we identified establishes that the influence of temporal context on risk is so fundamental and robust that it is visible even if existing risky decision-making studies and models are not designed to capture that influence. Conceptually similar recent studies have examined the implications of dynamic context effects for static models of value-based decision-making^38,42^, while others have begun to develop inherently contextual models of cognition and decision-making using frameworks like normalization^43–45^, sampling^36,46–49^, and others^10,50^. However, none of these models yet account for the trial-by-trial effects of previous outcomes on subsequent valuation and choices observed here. The development of sophisticated, contextually-sensitive models will be critical if future studies are to optimally tune their designs to further characterize and quantify the effects of context on valuation and action.

The creation of such models will also be indispensable to efforts to identify the underlying affective and neural correlates. While affect is clearly related to risky decision-making^2,4,19,51,52^, it is still unclear how affect may be related to the effects of context in the same domain. Theories have suggested a variety of possible ways affect could interact with or represent context, including that positive affect in a given moment guides future behavior to maintain that affect^13^; that affective moods may represent recent experiences in reinforcement learning^53^; or that affect may have an even broader and more fundamental role in representing a decision-maker’s context^54,55^. Given the recent finding that the central neural systems of valuation and choice may represent some contextual variables^10^, it seems likely that regions like the ventral striatum, amygdala, and ventromedial prefrontal cortex, which are deeply involved in decision-making^51,56,57^, are primary candidates for the neural mechanisms of context effects on risky decision-making.

Together, these findings suggest that risky monetary decision-making with moment-to-moment feedback is inherently dynamic and that a context-dependent, time-sensitive model is necessary for understanding temporal context effects underlying valuation and risky choice behavior. This critical insight will not only advance the study of risky monetary decision-making but may have real-world implications in settings where people may have different experiences (e.g. in aging, or across socioeconomic statuses), or think and feel differently about those experiences (e.g. in depression). Risky choices must be considered not only as a product of the individual decision-maker, but of what they have experienced.

## Method

### Data

We analyzed data collated across four previously published studies of risky monetary decision-making^2–5^. Each study had between 30 and 120 participants completing between 140 and 180 trials of a structurally identical risky monetary decision-making task (see Supplementary Material and Table S2 for a more in-depth discussion of the minor methodological variations across the four studies) in a fully-instructed laboratory setting (including detailed and guided task instruction, comprehension quizzes, and practice trials), producing high-quality incentive-compatible individual-level choice data. The complete dataset comprised a total of 64,953 binary choices made by a total of 234 participants (128 females, mean age = 23.4 (4.8), median age = 22; 207 missed trials were removed from analysis). To minimize confounding influences of the different manipulations and structure present in the four studies, portions of the data were removed from this analysis so that all data used reflected participants’ first experience with the task, in the absence of any external manipulation. To accomplish this, we removed all choices made on Day 2^2,4,5^, made with an emotion regulation-like strategy^2^, made under the influence of a medication^4^, or made after a stress manipulation^5^. The remaining data consisted of 23,373 choices made by a total of 151 participants (88 females), mean (sd) age of 23.4 (4.7) years old (median age = 22). All four studies were IRB approved and featured standard consent procedures. For more detailed information, see the individual studies^2–5^. The University of Denver’s Institutional Review Board determined that the current project did not require IRB review (January 18, 2019).

### Task

All studies had the same basic instruction and trial-level structure. Each began with detailed task instructions that were reviewed with the experimenter, a brief quiz to assess task comprehension, and a series of practice trials to ensure participants understood the task and were fully informed. In the main task, participants made a series of binary choices between risky gambles and guaranteed alternatives (see Fig. 1). The gamble always offered two potential outcomes with equal (50%) probability, while the guaranteed alternative delivered a single outcome with 100% probability (if chosen). Choice options were displayed for 2–4 s, followed by a response window of 2 s, then an interstimulus interval of 1 s before the choice outcome was realized for 1 s. Intertrial intervals varied between 1–3 s. The majority of trials were gain-loss trials in which potential risky gamble outcomes featured a gain amount ($2-$12) or a corresponding loss amount that was a negative multiple (×0.25-×2) of the gain amount. The remaining trial types were gain-only in which potential risky gamble outcomes featured a gain amount ($2-$30) or an outcome of $0, and a smaller guaranteed alternative ($1-$12). Every participant completed both gain-loss and gain-only trial types intermixed. At the end of the task, participants were paid a portion (10%) of trial outcomes relative to an initial endowment of $30, making all four studies incentive-compatible. The four studies varied slightly in total number of trials and blocks and the number of gain-loss and gain-only trials. See Supplementary Material and Table S2 for details on the minor variations across studies.

### Analysis: logistic regression models

To examine whether recent events influence subsequent risky choice behavior, we fit mixed effects logistic regression models to the risky binary choice data using the package “lme4” in R^26^. All generalized mixed effects models reported here are logistic regressions that use a linear combination of predictors and a binomial link function. We regressed binary choice on current choice options (including current risky gain, risky loss, and guaranteed alternative amounts), and one or more elements of previous events, including previous outcomes, previous decisions, and/or mean expected value of the previous choice options. When applicable, mean expected value on a given trial was calculated as [(0.5 × risky gain + 0.5 × risky loss) + (1 × guaranteed)]/2. In general, regressions included an intercept, current choice options, and previous choice outcomes/decisions/options as fixed effects and a random intercept for each participant. See Table S3 for lme4 code corresponding to each regression (models 1–9) in the Results.

We tested the output of model 7 in two ways: testing the entire model against model 1 with a likelihood ratio test (because model 1 is nested within model 7, that is, model 1 is a special case of model 7, in which the parameter estimates for previous risky gains, risky losses, and safe outcomes are equal) and testing the equality of specific pairs of coefficients with Wald Tests. The model comparison likelihood ratio test statistic was chi-square distributed with two degrees of freedom for the two extra regressors. The three Wald tests compared the coefficients estimated in model 7 for the effects of previous gains vs losses, previous gains vs guaranteed alternatives, and previous losses vs guaranteed alternatives. Tests were conducted using the “linearHypothesis” function in the package “car” in R^58^, and were chi-square distributed with 1 degree of freedom. A Wald test was also used to test the effects of previous gain outcomes against previous loss outcomes in model 8 in an identical procedure.

### Analysis: non-linear prospect theory plus model

We fit a modified version of a 4-parameter prospect theory-inspired model of non-linear processes underlying valuation and choice (Prospect Theory Plus, PT + )^10^. Prospect Theory and related models, like PT + , are a class of nonlinear models that are both commonly used on, and acknowledged to provide a good fit to risky decision-making data^1,27,28,33,34^. In brief, the PT + model transformed objective values to subjective values in utility functions, which were then used to compute the likelihood of making a risky choice using a softmax function. The model contains four parameters capturing four distinct decision-making processes in three equations. The subjective values of risky gains, risky losses, and guaranteed alternatives were calculated with Eqs. (1) & (2):

1 $${\rm{u}}({x}^{+})={\rm{p}}({x}^{+}){({x}^{+})}^{\rho }$$

2 $${\rm{u}}({x}^{-})={\rm{p}}({x}^{-})\times -\lambda \times {(-{x}^{-})}^{\rho }$$

In these equations, p(*x*) is the probability of receiving the value, *x*. Consider an example choice between a gamble with a risky gain of + $12 and a risky loss of -$10, and a guaranteed alternative of $0. The utility calculations for the risky gain, risky loss, and guaranteed alternative would be as follows for someone with parameters *ρ* = 0.8 and *λ* = 1.5:

$$\begin{array}{c}{\rm{u}}({\rm{risky}}\,{\rm{gain}})=0.5\times {(+\$12)}^{0.8}=+\,3.65\\ {\rm{u}}({\rm{risky}}\,{\rm{loss}})=0.5\times -\,1.5\times {(-(-\$10))}^{0.8}=-\,4.73\\ {\rm{u}}({\rm{guaranteed}}\,{\rm{alternative}})=1\times {(\$0)}^{0.8}=0\end{array}$$

The parameter *ρ*, present in Eqs. (1) and (2), captures diminishing sensitivity to changes in value as the absolute value increases, represented as curvature of the utility function. For choices exclusively in the gain domain, when *ρ* < 1, an individual is risk averse, *ρ* = 1 indicates risk neutrality, while *ρ* > 1 indicates risk seeking. In the loss domain, the effect of *ρ* on risk-taking is reversed: *ρ* < 1 indicates risk seeking for losses, *ρ* = 1 indicates risk neutrality, and *ρ* > 1 indicates risk aversion for losses. This reversal is often called the reflection effect^1^.

The parameter* λ*, present in Eq. (2), captures loss aversion as a multiplicative weight on losses as compared to gains. When *λ* = 1, gains and losses are valued equally (“gain-loss neutral”), while *λ* > 1 indicates the overvaluation of losses compared to gains (“loss averse”), and *λ* < 1 indicates the undervaluation of losses compared to gains (“gain seeking”).

The third equation is the logit, or softmax function which integrates the utility of the gamble (i.e. the sum of the utility of the risky gain and risky loss) and the guaranteed alternative, and a value-independent decision bias (*db)* to calculate the probability (between 0 and 1) of accepting the gamble:

3 $${\rm{p}}({\rm{a}}{\rm{c}}{\rm{c}}{\rm{e}}{\rm{p}}{\rm{t}}\,{\rm{g}}{\rm{a}}{\rm{m}}{\rm{b}}{\rm{l}}{\rm{e}})={(1+{{\rm{e}}}^{\text{-}\mu \times ({\rm{u}}({\rm{g}}{\rm{a}}{\rm{m}}{\rm{b}}{\rm{l}}{\rm{e}})\text{-}{\rm{u}}({\rm{g}}{\rm{u}}{\rm{a}}{\rm{r}}{\rm{a}}{\rm{n}}{\rm{t}}{\rm{e}}{\rm{e}}{\rm{d}})\text{-}db)})}^{\text{-}1}$$

Equation (3) contains two parameters. First, mu (*μ*) quantifies the extent to which an individual’s binary choices respond to changes across trials in the value of the gamble as compared to the guaranteed alternative. A *μ* of 0 indicates complete randomness across choices, whereas increasing values of *μ* indicate greater consistency across choices. Second, the parameter *db* represents a value-independent decision bias toward or away from risk-taking, captured as a leftward or rightward shift in the softmax function. *db* < 0 indicates a bias to gamble (softmax function shifts left on *x*-axis) and *db* > 0 indicates a bias to not gamble (softmax function shifts right on *x*-axis). For more on decision bias, see reference^10^.

The parameters* ρ* and *λ* are value-dependent parameters, as they affect the transformation of objective value to subjective value. The parameter *db* is value-independent as it simply affects a propensity for action (risk-taking) without regard to the values present on a given trial. *μ* is an intermediate kind of parameter, as it links values to action.

### Analysis: hierarchical bayesian model-fitting

To estimate the parameter values of PT+, we used Markov-Chain Monte Carlo (MCMC) sampling techniques in a hierarchical Bayesian framework. This approach explicitly fits individual-level parameters (e.g. participant 1’s decision bias, participant 2’s decision bias, etc.) and group-level parameters (e.g. the group-level mean and standard deviation describing the distribution of decision bias parameter values across individuals), thereby capturing both individual variability and the similarity across individuals to maximal statistical effect while simultaneously reducing the impact of outliers. This kind of approach has been used successfully to model risky decision-making in both our own^5^ and others’^33,34^ research.

To model each of the four main parameters of PT+ over time, we modeled their initial value and an update parameter that controlled how much previous decision outcomes shifted that parameter on subsequent trials. To do this, we altered PT+ to include four additional updating parameters (four *δ*^*θ*^ terms, i.e. *δ*^*ρ*^, *δ*^λ^, *δ*^*μ*^, *δ*^*db*^). Thus, each of the four main PT+ parameters were modeled for a given participant as follows:

4 $${{\rm{\theta }}}_{1}={{\rm{\theta }}}_{{\rm{initial}}}$$

5 $${{\rm{\theta }}}_{{\rm{t}}}={{\rm{\theta }}}_{{\rm{t}}-1}+{{\rm{outcome}}}_{{\rm{t}} \mbox{-} 1}\times {\delta }^{\theta }$$

Equation (4) represents the baseline value for each parameter (*λ*, *ρ*, *μ* and *db* any of which is represented by θ) on the first trial for a given participant. Equation (5) describes the value of each parameter on a given trial *t* (*θ*_*t*_ for *t* > 1) as a function of the previous value of that parameter (*θ*_*t*-*1*_), the outcome on the previous trial (outcome_*t*-*1*_, linearly scaled to be between 0 and 1), and the update parameter (*δ*^*θ*^).

All participants’ individual-level parameters (each of *ρ*, *λ*, *μ*, *db, δ*^*ρ*^, *δ*^*λ*^, *δ*^*μ*^, and *δ*^*db*^) were normally-distributed around a group-level mean and standard deviation for that parameter. When examining model output, we therefore focused analysis on estimates of the group-level mean parameters (e.g. *λ*_*mean*_).

For each parameter, we calculated 95% confidence intervals (CIs) for the posterior distribution of samples for that parameter. In the case of the update parameters, we examined whether each 95% CI contained zero. If the CI did not contain zero, we could conclude, with 95% confidence, that the true value of that update parameter was not zero, suggesting that previous outcomes had indeed influenced the value of that parameter of PT+ over time.

See Supplementary Materials for more information, including priors for the group-level parameters and detailed explanation of the mechanics of model estimation and the use of mathematical transformations to gently implement parameter bounds. The full Stan model code is also available on the Open Science Framework: https://osf.io/npd54/.

## Supplementary information

Supplementary Information.

## Data Availability

The complete datasets analyzed in the current study, along with the Stan code for the hierarchical Bayesian model of PT+ are available on the Open Science Framework at https://osf.io/qxa9h/.
