# Supplementary material for: Quantifying the immediate computational effects of preceding outcomes on subsequent risky choices
Source: Sci Rep. 2020 Jun 18;10:9878. doi: 10.1038/s41598-020-66502-y (PMC7303130; doi:10.1038/s41598-020-66502-y)
Supplement: Supplementary file 1 — Supplementary Information. [file 41598_2020_66502_MOESM1_ESM.pdf]

Quantifying the immediate computational effects of preceding outcomes on subsequent risky choices

Hayley R. Brooks, Peter Sokol-Hessner

SUPPLEMENTARY MATERIAL

Study Details

The four studies included in this analysis had the same basic instructions and task structure including the initial endowment, the timing of decision and feedback events on each trial, the possible monetary amounts, and the task-related payment. The studies varied by participation fee ($15-$20), number of trials (140-180), number of days (1-2), and external manipulation. For this analysis, we excluded choices made under any experimental manipulation and on day 2 to capture participants' first experience with the risky monetary decision-making task. In Study 1, 30 participants switched between two cognitive reappraisal strategies ("regulate" and "attend") during the risky monetary choice task[1]. In the "attend", or baseline condition, participants were instructed to think about each choice independent of the other choices. In the "regulate" condition, participants were instructed to think about their choices as a portfolio ("you win some, you lose some"). Study 2 (N=37) involved no experimental manipulations during the risky choice task[2]. Study 3 was a two-day, double-blind, placebo-controlled, within-subjects study in which 47 participants were given the beta-blocker propranolol or a placebo prior to the risky monetary choice task[3]. In Study 4, a two-day, within-subjects study, 120 participants completed a cold-pressor task or a control manipulation (a warm-water bath) with equal probability prior to the risky monetary decision-making task on each day[4]. In total, there was 64,953 choices across 234 participants. For this analysis, we excluded the choices made in the "regulate" condition (4,200 trials from Study 1), in the propranolol condition (3,432 trials from Study 3), in the cold-pressor condition (8,968 trials from Study 4), and on day 2 (an additional 7,013 trials from Study 3 and 17,967 additional trials from Study 4), leaving a total of 23,373 trials across 151 participants. See Table S1 for summaries of the demographic and methodological differences across the four studies.

Additional MCMC Estimation Details

The Stan model code is available on the Open Science Framework: https://osf.io/npd54/

Priors

Sampling priors were selected to be uninformative as possible and were normal (mean, standard deviation), uniform (lower limit, upper limit), or cauchy (location, scale) distributions (see Table S1 below). Parameters were sampled in a different space than they were applied. A transformation was used to convert sampled values to applied values (see section below, *Transformation*)

parameter	Group Mean prior	Group SD prior
λ	Normal(0,30)	Cauchy(0,2.5)
ρ	Normal(0,30)	Cauchy(0,2.5)
μ	Uniform(0,30)	Cauchy (0,2.5)
db	Normal(0,30)	Cauchy(0,2.5)
δ^{λ}	Normal(0,10)	Cauchy(0,2.5)
δ^{ρ}	Normal(0,10)	Cauchy(0,2.5)
δ^{μ}	Normal(0,10)	Cauchy(0,2.5)
δ^{db}	Normal(0,10)	Cauchy(0,2.5)

Table S1: Priors for the group-level mean and standard deviation for each of the main parameters in the prospect theory plus model.

Transformations

For stability of estimation, the MCMC model transformed the sampled baseline parameters λ, ρ, and μ before applying them to the data in manner identical to that used previously[4] and similar to approaches used by others[5,6], in effect implementing a lognormal structure for those parameters. These transformations served to gracefully implement the minimum number of practical bounds on parameter values, without which models would experience numerical faults (overflow; impossible values) that would prevent successful estimation. See *Transformation Rationale* below for more discussion. In all cases, the values discussed in the text and in plots reflect the applied values of these parameters (i.e. after the transformation).

First baseline values for the PT+ parameters λ, ρ, and μ, all of which have theoretical lower bounds of 0, were sampled in unbounded 'sampling' space (bounds of [-infinity, +infinity]). To transform from sampling space to 'applied' space, these unbounded values were passed through an exponential (e.g. if the sampled value was R, exp(R) gave the applied value, ρ). An exponential transform produces strictly positive values of ρ for all real values of R (that is, ρ is bounded [0, +infinity]), thereby meeting the basic requirement that values of λ, ρ, and μ, be above 0. All effect sizes and plots reflect the applied (transformed, bounded) values of the parameters (that is, ρ not R).

The exponential transform was not applied to the decision bias parameter, which is theoretically unbounded.

Second, the update parameters (i.e. the δ^{θ} parameters), were transformed using individual softmax-based functions to gracefully constrain parameters between lower and upper bounds

symmetric around zero, while allowing sampling to occur smoothly in unbounded space. The softmax equations were built so adjustment terms in 'applied' space thus had lower/upper bounds of [-1,+1] (*db* and λ), and [-0.25, +0.25] (ρ and μ). All effect sizes reflect values in these applied (transformed, bounded) spaces.

In all cases, the final 95% confidence intervals for all parameters did not approach their respective bounds, suggesting that these bounds, while effective in enabling model estimation, did not interfere with identification of the most likely values of these parameters. While one cannot definitively state that there are no values of the parameters outside these bounds more likely than those we sampled, it is also not possible to identify them, as model estimation is not stable without these reasonable, psychologically plausible, and commonly-used bounds.
The model was coded in Stan (see Methods) – for complete model code, including all aspects of parameter transformations, see https://osf.io/npd54/.

Transformation rationale

We have previously published this hierarchical Bayesian implementation of prospect theory[4], which is structurally similar to that used by others[5,6]. In essence, in this approach the 'sampled' space of the parameters for rho, lambda, and mu are unbounded (i.e. with bounds of [-infinity, +infinity]), but the final parameter values applied to data (what we call "applied space"; after the use of the exponential, see above) are bounded [0, +infinity]. We use the exponential to implement a lower bound of 0 for three of our four parameters (rho, lambda, and mu), for two main reasons:

1) It smoothly implements a lower bound of 0 (which is required for rho, lambda, and mu), while leaving the parameters unconstrained in the positive direction, as they have no theoretical upper bound (even if their plausible, expected, and psychologically-likely values are generally lower).

2) It gracefully allows the summation of independent terms contributing to the value of a parameter on a given trial in unbounded, 'sampled' space (e.g. the effect of previous outcomes on the loss aversion parameter) while preventing those summations from under-flowing.

Interaction between the priors and transformations

As a result of the prior distributions going through an exponential to generate softly-bounded final parameters, the priors favored lower values for each of the parameters. This approach was deemed reasonable for three reasons. First, lower values of these parameters are indeed psychologically most likely (e.g. rho values tend to be reported between .5 and 1.5; lambda values between 0.5 and 4). Second, the priors, after being put through the exponential transformation are still broad and relatively uninformative. Lastly, our MCMC sampling procedure discarded the first 5,000 samples (50%) of each of the twenty chains, effectively eliminating the influence of the selected priors on the final sampled posterior distributions.

1. Sokol-Hessner, P. *et al. Thinking like a trader selectively reduces individuals' loss aversion*. *PNAS* **31**, (2009).
2. Sokol-Hessner, P., Hartley, C. A., Hamilton, J. R. & Phelps, E. A. Interoceptive ability predicts aversion to losses. *Cogn. Emot.* **29**, 695–701 (2015).
3. Sokol-Hessner, P. *et al.* Determinants of Propranolol's Selective Effect on Loss Aversion. *Psychol. Sci.* **26**, 1123–1130 (2015).
4. Sokol-Hessner, P., Raio, C. M., Gottesman, S. P., Lackovic, S. F. & Phelps, E. A. Acute stress does not affect risky monetary decision-making. *Neurobiol. Stress* **5**, 19–25 (2016).
5. Nilsson, H., Rieskamp, J. & Wagenmakers, E.-J. Hierarchical Bayesian parameter estimation for cumulative prospect theory. *J. Math. Psychol.* **55**, 84–93 (2011).
6. Scheibehenne, B. & Pachur, T. Using Bayesian hierarchical parameter estimation to assess the generalizability of cognitive models of choice. *Psychon. Bull. Rev.* **22**, 391–407 (2015).

Table S2. Characteristics of the previously published studies in the current analysis.

Study #		1	2	3	4
**Title**		Thinking like a trader selectively reduces individuals' loss aversion.	Interoceptive ability predicts aversion to losses.	Determinants of propranolol's selective effect on loss aversion.	Acute stress does not affect risky monetary decision-making.
**Authors**		Sokol-Hessner, P., Hsu, M., Curley, N.G., Delgado, M.R., Camerer, C.F., Phelps, E.A.	Sokol-Hessner, P., Hartley, C.A., Hamilton, J.R., Phelps, E.A.	Sokol-Hessner, P., Lackovic, S.F., Tobe, R.H., Camerer, C.F., Leventhal, B.L., Phelps, E.A.	Sokol-Hessner, P., Raio, C.M., Gottesman, S.P., Lackovic, S.F., Phelps, E.A.
**Year**		2009	2015a	2015b	2016
**Journal**		PNAS	Cognition and Emotion	Psychological Science	Neurobiology of Stress
**N**		30	37	47	120
**Sex**		11 females; 19 males	25 females; 12 males	22 females; 25 males	64 females; 56 males
**Age, mean(sd)**		22(3) years	24.32(4.6) years	26.6(5.1) years	22.4(4.5) years
**Trials per participant**		140 (120 gain-loss; 20 gain-only)	180 (150 gain-loss; 30 gain-only)	150 (120 gain-loss; 30 gain-only)	
**Days**		1		2	
**Incentive structure**	**Subject fee**	$15		$20/hr	$15/hr
	**Endowment**	$30			
	**Payout**	outcomes on 10% of randomly selected trials			
**Timing of task events**		4s viewing window, 2s response period, 1s ISI, 1s outcome, 1-3s ITI	2s viewing window, 2s response window, 1s ISI, 1s outcome, 1-3s ITI		
**Experimental methodology**		One-day, within-subjects study. Participants completed a risky monetary choice task while switching between two cognitive reappraisal (attend vs. regulate) strategies.	One-day, within-subjects study. Participants completed a heartbeat detection task (as a measure interoception) followed by the risky monetary gambling task.	Double-blind, placebo-controlled, within-subjects study. On each day, participants were administered either propranolol or a placebo prior to completing risky monetary choice task.	Fully-crossed, two-day, within-subjects study. Participants completed a cold pressor task or a control manipulation with equal probability prior to making a series of risky monetary choices. Acute stress was measured by salivary cortisol samples.
**Exclusion criteria for this analysis**		Choices made during "reappraisal" condition.	NA	Choices made during the adminsitration of propranolol and on day 2.	Choices made during the stress condition and on day 2.
**Missed trials**		0	19	32	42

Table S3. Generalized linear modeling results using the "lme4" package (R version 3.5.0; “lme4” version 1.1-21).

Model 1 = glmer(choice ~ 1 + risky gain amount(t) + risky loss amount(t) + safe amount(t) + outcome amount(t-1) + (1|Subject ID), data, family = "binomial")

Model 1 results

AIC	BIC	logLik	deviance	df.resid
21289	21337.3	-10638.5	21277	23216

Fixed effects

	Estimate	Std Error	Z value	Pr(>|z|)
intercept	-0.067457	0.104245	-0.647	0.518
risky gain amount(t)	0.299218	0.007126	41.99	< 2e-16 ***
risky loss amount (t)	0.397172	0.006532	60.804	< 2e-16 ***
safe amount (t)	-0.590955	0.014532	-40.666	< 2e-16 ***
outcome amount(t-1)	-0.030859	0.003092	-9.979	< 2e-16 ***

Model 2 = glmer(choice ~ 1 + risky gain amount(t) + risky loss amount(t) + safe amount(t) + choice(t-1) + (1|Subject ID), data, family = "binomial")

Model 2 results

AIC	BIC	logLik	deviance	df.resid
21377.5	21425.8	-10682.7	21365.5	23216

Fixed effects

	Estimate	Std Error	Z value	Pr(>|z|)
intercept	-0.134037	0.105756	-1.267	0.205003
risky gain amount(t)	0.299066	0.007109	42.066	< 2e-16 ***
risky loss amount (t)	0.396355	0.006520	60.795	< 2e-16 ***
safe amount (t)	-0.590458	0.014502	-40.717	< 2e-16 ***
choice(t-1)	-0.068814	0.018671	-3.686	0.000228 ***

Model 3 = glmer(choice ~ 1 + risky gain amount(t) + risky loss amount(t) + safe amount(t) + mean EV(t-1) + (1|Subject ID), data, family = "binomial")

Model 3 results

AIC	BIC	logLik	deviance	df.resid
21350.4	21398.7	-10669.2	21338.4	23216

Fixed effects

	Estimate	Std Error	Z value	Pr(>|z|)
intercept	-0.088786	0.103914	-0.854	0.393
risky gain amount(t)	0.299548	0.007121	42.066	< 2e-16 ***
risky loss amount (t)	0.39585	0.006517	60.744	< 2e-16 ***
safe amount (t)	-0.59061	0.014521	-40.674	< 2e-16 ***
mean EV (t-1)	-0.032006	0.00505	-6.337	2.34e-10 ***

Model 4 = glmer(choice ~ 1 + risky gain amount(t) + risky loss amount(t) + safe amount(t) + mean EV(t-1) + choice(t-1) + outcome amount(t-1) + (1|Subject ID), data, family = "binomial")

Model 4 results

AIC	BIC	logLik	deviance	df.resid
21288.7	21353.2	-10636.4	21272.7	23214

Fixed effects

	Estimate	Std Error	Z value	Pr(>|z|)
intercept	-0.078854	0.105503	-0.747	0.4548
risky gain amount(t)	0.299485	0.00713	42.004	< 2e-16 ***
risky loss amount (t)	0.397197	0.006534	60.787	< 2e-16 ***
safe amount (t)	-0.591271	0.014536	-40.676	< 2e-16 ***
mean EV(t-1)	-0.001755	0.006312	-0.278	0.7809
choice(t-1)	-0.037183	0.019637	-1.893	0.0583 .
outcome amount(t-1)	-0.029276	0.003728	-7.853	4.08e-15 ***

Model 5 = glmer(choice ~ 1 + risky gain amount(t) + risky loss amount(t) + safe amount(t) + outcome amount(t-1) + outcome amount(t-2) + outcome amount(t-3) + (1|Subject ID), data, family = "binomial")

Model 5 results

AIC	BIC	logLik	deviance	df.resid
20941.7	21006	-10462.8	20925.7	22912

Fixed effects

	Estimate	Std Error	Z value	Pr(>|z|)
intercept	-0.066689	0.105297	-0.633	0.5265
risky gain amount(t)	0.300642	0.00716	41.961	< 2e-16 ***
risky loss amount (t)	0.397693	0.006589	60.361	< 2e-16 ***
safe amount (t)	-0.592879	0.014614	-40.569	< 2e-16 ***
outcome amount(t-1)	-0.031339	0.003114	-10.064	< 2e-16 ***
outcome amount(t-2)	-0.007769	0.003050	-2.547	0.0109 *
outcome amount(t-3)	-0.003964	0.003057	-1.296	0.1948

Model 6 = glmer(choice ~ 1 + risky gain amount(t) + risky loss amount(t) + safe amount(t) + outcome amount (t-1) + outcome valence(t-1) + (1|Subject ID), data, family = "binomial")

Model 6 results

AIC	BIC	logLik	deviance	df.resid
21287.8	21344.2	-10636.9	21273.8	23215

Fixed effects

	Estimate	Std Error	Z value	Pr(>|z|)
intercept	-0.066248	0.104291	-0.635	0.525
risky gain amount(t)	0.299004	0.007126	41.96	< 2e-16 ***
risky loss amount (t)	0.397148	0.006533	60.795	< 2e-16 ***
safe amount (t)	-0.590608	0.014532	-40.642	< 2e-16 ***
outcome amount (t-1)	-0.037835	0.005038	-7.509	5.95e-14 ***
outcome valence (t-1)	0.079089	0.044875	1.762	0.078 .

Model 7 = glmer(choice ~ 1 + risky gain amount(t) + risky loss amount(t) + safe amount(t) + win amount(t-1) + loss amount(t-1) + safe amount(t-1) + (1|Subject ID), data, family = "binomial")

Model 7 results

AIC	BIC	logLik	deviance	df.resid

21291.8	21356.2	-10637.9	21275.8	23214
		Fixed effects		
	Estimate	Std Error	Z value	Pr(>|z|)
intercept	-0.064496	0.104951	-0.615	0.538861
risky gain amount(t)	0.299152	0.007126	41.979	< 2e-16 ***
risky loss amount (t)	0.397241	0.006533	60.802	< 2e-16 ***
safe amount (t)	-0.590842	0.014532	-40.659	< 2e-16 ***
win amount(t-1)	-0.033083	0.003973	-8.327	< 2e-16 ***
loss amount(t-1)	-0.02797	0.007819	-3.577	0.000348 ***
safe amount(t-1)	-0.023994	0.008558	-2.804	0.005053 **

Model 8 = glmer(choice ~ 1 + risky gain amount(t)+ risky loss amount(t) + safe amount(t) + gain amount(t-1)*choice(t-1) + loss amount(t-1) + (1|Subject ID), data, family = "binomial")

		Model 8 results		
AIC	BIC	logLik	deviance	df.resid
21290.2	21362.7	-10636.1	21272.2	23213
		Fixed effects		
	Estimate	Std Error	Z value	Pr(>|z|)
intercept	-0.0899602	0.106447	-0.845	0.398
risky gain amount(t)	0.2994444	0.00713	42	< 2e-16 ***
risky loss amount (t)	0.3972658	0.006533	60.808	< 2e-16 ***
safe amount (t)	-0.5912799	0.014536	-40.676	< 2e-16 ***
gain amount(t-1)	-0.0272841	0.0049348	-5.529	3.22e-08 ***
choice(t-1)	-0.0458073	0.024232	-1.89	0.0587 .
loss amount(t-1)	-0.0357708	0.008868	-4.034	5.49e-05 ***
gain amount(t-1)*choice(t-1)	-0.0008651	0.004943	-0.175	0.8611

Model 9 = glmer(choice ~ 1 + risky gain amount(t)*outcome amount(t-1) + risky loss amount(t)*outcome amount(t-1) + safe amount(t)*outcome amount(t-1) + (1|Subject ID), data, family = "binomial")

Model 8 results

AIC	BIC	logLik	deviance	df.resid
21278.6	21351.1	-10630.3	21260.6	23213

Fixed effects

	Estimate	Std Error	Z value	Pr(>|z|)
intercept	-0.08573	0.1045	-0.82	0.4120
risky gain amount(t)	.299348	0.007348	40.738	< 2e-16 ***
risky loss amount (t)	0.394433	0.0066	59.761	< 2e-16 ***
safe amount (t)	-0.590369	0.014989	-39.386	< 2e-16 ***
outcome amount(t-1)	-0.45562	0.205493	-2.217	0.02660 *
risky gain amount(t)* outcome amount(t-1)	0.012088	0.04071	0.297	0.76653
risky loss amount(t)* outcome amount(t-1)	0.1019	0.03271	3.128	0.00176**
safe amount(t)* outcome amount(t-1)	-0.056176	0.082578	-0.68	0.49633
